# Coping Strategies, Creativity, Social Self-Efficacy, and Hypercompetitiveness in Gambling Behaviors: A Study on Male Adolescent Regular Gamblers

**DOI:** 10.3389/fpsyg.2020.01722

**Published:** 2020-07-21

**Authors:** Alessia Passanisi, Giulio D’Urso, Adriano Schimmenti, Stefano Ruggieri, Ugo Pace

**Affiliations:** Faculty of Human and Social Science, UKE – Kore University of Enna, Enna, Italy

**Keywords:** gambling, adolescents, coping strategies, self-efficacy, creativity, hypercompetitiveness

## Abstract

The purpose of this research was to explore the cognitive and personality characteristics of male adolescent gamblers. Participants were 273 teenage males (*M* = 18.04, *SD* = 2.10) attending betting centers in Sicily, who completed self-report questionnaires on gambling, creativity, perceived social self-efficacy, hypercompetitiveness, and coping strategies. Pathological gamblers reported higher levels of avoidant coping strategies than occasional gamblers. They also scored higher on hypercompetitiveness than both occasional and problem gamblers. Further, problem gamblers scored higher than occasional gamblers on the complexity domain of creative personality. Finally, poor perceived social self-efficacy, higher levels of avoidant coping, and hypercompetitiveness predicted pathological gambling. Theoretical, psycho-educational, and clinical implications are discussed.

## Introduction

Pathological gambling (PG) belongs to the diagnostic class of substance-related and addictive disorders. Indeed, in line with DSM-5 (American Psychiatric Association, 2013), gambling disorder is considered a non-substance-associated addictive behavior and may be defined as a pattern of insistent and repeated gambling behavior leading to extensive clinical impairments: people suffering from gambling disorder display symptoms such as a desire to gamble with increasing amounts of money, feelings of anxiety or irritability connected to the inability to play, risking bonds and career because of gambling, and other symptoms, including episodes of craving, such as an uncontrollable desire to play, and gambling when feeling distressed. Several studies underline how PG is a problem situation more usual among boys than girls (Ellenbogen et al., 2007; Pace and Passanisi, 2018).

Specifically, men bet and hazard more and have more difficulty related to gambling during adolescence than girls (Calado et al., 2017), but this “gender gap” in gambling involvement decreases during adulthood.

In addition, this problem affects both young people and adults in terms of incidence. Most gambling activities are legally restricted to adults in the majority of countries, but adolescent gambling is not infrequent. Adolescents occasionally, although they bet and hazard less, show more severe episodes than adults (Bastiani et al., 2013). Specifically, the current generation of adolescents and young adults constitutes a susceptible age group because they have grown up in a time with widespread gambling opportunities (Gupta and Derevensky, 2004) that, for a small minority of youth, particularly males, instead of constituting a recreational activity can lead to severe negative outcomes (Calado et al., 2017), such as poor academic performance, injury, and dating violence (Thombs et al., 2009; Afifi et al., 2010). Given the great social costs of PG, it is important to explore those processes and risk factors that lead adolescents, statistically, more boys than girls, from gambling to significantly more structured PG in adulthood.

Several studies suggest that the number of adolescents who engage in risky behavior is constantly growing as, in this life stage, they tend more often to consider themselves invulnerable and lack knowledge about the negative consequences of such behaviors (e.g., Grant and Kim, 2005; Derevensky et al., 2010). The literature on PG has attempted to create different player profiles of gamblers. Indeed, Abbott et al. (1995) classified gamblers as “excessive” or “normal” on the basis of the time spent gambling, expense, and number of trips to the gaming sites. Gupta and Derevensky (1998) differentiated social players from problematic and pathological players, thus conceiving gambling behaviors along a continuum between normality and pathology (i.e., when the game induces the characteristics of chronic stress). According to Blaszczynski and Nower (2002), basically, there are three kinds of PGs. These comprise emotionally vulnerable subtypes, characterized by cognitive distortions and poor coping strategies.

In other words, if there are individual differences leading to different kinds of vulnerability to PG, then it could be relevant to better explore the categories of gamblers considering those individual variables of a social-cognitive and personality nature that can represent risk factors for gambling behaviors.

In this regard, some studies highlight how dysfunctional coping strategies can be involved in adolescent gambling (Bergevin et al., 2006; Shead et al., 2010; Dixon et al., 2016). According to extensive literature, adolescents may use various coping strategies: problem-focused strategies (e.g., striving to modify an event and aiming to reduce the stressful condition); emotion-focused strategies, which aim to diminish the emotional burden connected to the perceived stress (e.g., detachment from the situation, seeking social support); and avoidance-oriented strategies, by which the person tries to escape from the stressful situation (Lazarus, 1983; Roth and Cohen, 1986; Nigro, 1996). In particular, Sharpe and Tarrier (1993) highlight that coping strategies are fundamental mechanisms that separate “controlled” from “excessive” gamblers. Exploratory studies suggest that adolescent gamblers who excessively play exhibit more emotionally based, avoidance, and distraction-oriented coping styles (Gupta et al., 2000, 2004; Nower et al., 2004; Verner-Filion et al., 2014; Casey et al., 2017). Moreover, Bergevin et al. (2006) found that teenagers with gambling problems exhibit less task-centered coping levels as well as more avoidance-focused strategies. Furthermore, problematic male players would use emotion-focused coping strategies more than women.

Another psychological variable leading adolescents to PG could be hypercompetitiveness. This attitude denotes a deep need by people to win by competing to keep or to increase feelings of self-worth and self-esteem with a particular tendency toward aggression, control, denigration, and manipulation of other people (Ryckman et al., 1997). A few studies highlight how pathological gamblers show higher ranks of hypercompetitive attitudes due to obsession with achievement of goals and success (Walters, 1994; Burger et al., 2006; Passanisi et al., 2019). Because hypercompetitiveness is an intergroup construct, pathological gamblers, in this sense, need each other to feel powerful and strong to be able to achieve success. This attitude can be considered a cultural style in which the characteristics of ruthlessness are seen as positive and, therefore, as adaptive traits.

A few other studies show a connection among creativity and gambling (Pascual-Leone et al., 2011). The creative personality defines a person who can solve problems, develop products, or formulate new questions in a manner that is first considered original but ends up being accepted in a particular cultural environment (Gardner, 1988). Creativity is consequently a form of divergent and unconventional thought (Guilford, 1950, 1967), which can produce unusual responses. In this sense, creativity can be connected to gambling because it helps individuals to create a great number of original solutions for a given problem. This means that those who are at-risk gamblers may display some relevant differences in the way that they manage tasks, which may be linked to a larger factor of being exploration-oriented or unconventional. A recent study carried out on a sample of university students showed that at-risk gamblers had high levels of creativity, whereas non-players and problem gamblers showed equally low levels of creativity (Pascual-Leone et al., 2011).

Finally, research highlights that people’s beliefs about their self-efficacy in managing events influence choices, aspirations, levels of effort, perseverance, vulnerability to stress, and in general the quality of performance (Bandura and Cervone, 1983; Kaur et al., 2006). Moreover, literature shows that individuals with low levels of self-efficacy are more prone to undertake addictive behaviors and that addictive behaviors positively correlate with lower social interactions, lower self-esteem, isolation, and depression (Kraut et al., 1998; Ko et al., 2005). Thus, self-efficacy can be considered a critical protective factor for the etiology of behavioral addictions, such as PG (Sylvain et al., 1997; Raylu and Oei, 2002; Hyde et al., 2008). In particular, the study conducted by Jeong and Kim (2011) suggests that perceived social self-efficacy, one facet of actual social abilities referring to a willingness to start conduct in social environments (Sherer and Adams, 1983; Smith and Betz, 2000) and to individuals’ perception that they are capable of starting public interaction as well as making new friendships (Gecas, 1989), diminished with implemented adolescent addictive behaviors, in particular related to gambling. Conversely, individuals with high levels of social self-efficacy were less at risk of falling into addictive tendencies. Therefore, lack of social self-efficacy would be the launch pad toward the implementation of compensatory maladaptive behaviors that may result in the development of a behavioral craving (Kardefelt-Winther et al., 2017). Studying perceived social self-efficacy concerning gambling can broaden the description of the psychological variables related to this phenomenon.

In line with the aforementioned theoretical premises, the aim of the present research was to investigate certain cognitive features (i.e., perceived social self-efficacy, hypercompetitiveness attitude, creativity, and coping strategies) that may represent protective or risk factors of PG in a male adolescent population by assessing the differences between three gambling categories: occasional gamblers, problem gamblers, and pathological gamblers.

## Methods

### Participants and Procedure

Participants were 273 male adolescents and young adults aged 15–19 (*M*_age_ = 18.04, *SD* = 2.10) contacted in betting centers in Sicily, even though minors under 18 are not allowed to bet in Italian social fabric, between February 2019 and February 2020.

The adolescents were informed about the research objectives while they were in the game centers. After their written informed consent was obtained, they were requested to complete an anonymous battery of self-report tools to evaluate creativity, perceived social self-efficacy, coping styles, and gambling. The group of participants represents a convenience sample because we recruited the adolescents and young adults who were present in the main centers of the territory. The adolescents who agreed to participate in the study were also informed about available treatment centers to favor their contact with health services.

During the administration of the questionnaires, the participants were left free to abandon the administration at any time. Furthermore, given the particular legal situation, it was not possible to identify underage subjects, nor obviously ask their parents for informed consent. If we had to proceed with the usual procedures for identifying and parental consent of minors, we should have given up the research. We believe, on the contrary, that despite the identification difficulties, carrying out this study was important for the prevention of illicit behaviors during adolescence that can be pathologically structured in adulthood.

The research processes explained in this manuscript adhered to the ethical norms for research and were accepted by the internal review board (IRB) for psychological research of the UKE – Kore University of Enna (approval code: UKE-IRBPSY-04.20.01).

### Measures

#### The South Oaks Gambling Screen

The South Oaks Gambling Screen (SOGS; Lesieur and Blume, 1987; Marazziti et al., 2014) is an 18-item, self-report tool that came from DSM criteria for PG. It is split into two parts: the first five items give information on the kind of gambling (e.g., bet on horses, play bingo for money, play cards, etc.) and on related topics [e.g., “Have you ever quit gambling for a period of time?” “What is the largest amount of money you have ever gambled on any one day?” “Are there some people in your life who have (or had) a gambling problem?”]. Items from 6 to 18 concern information on the occurrence of behaviors linked to gambling (e.g., “When you play the game of chance and lose, how often do you return the next day to try to win the amount lost?” “Have you ever gambled more than you wanted?”). Adding up the number of items with an “at-risk” response gives the scores of SOGS. The first five questions are not considered for the total mark. As for the remaining items, some of them can be calculated more than once. The Cronbach’s alpha value is 0.702.

#### The Test of Creative Thinking

The Test of Creative Thinking consists of 50 items evaluating four levels of Williams (1994) classification for original thinking: curiosity, imagination, complexity, and risk taking. This measure is administered to children and adolescents; each answer obtains a score from −1 to 2 points (from almost always false to almost always true). For the current research, we only administered the “curiosity” (e.g., I like trying many new things; Cronbach alpha value is 0.74) and “complexity” scales (e.g., I like trying to solve a problem even when there is not a single solution; I like “different” things; Cronbach’s alpha value is 0.71).

#### The Perceived Social Self-Efficacy Scale

The Perceived Social Self-Efficacy Scale (PSSE; Smith and Betz, 2000; Di Giunta et al., 2010) measures individuals’ beliefs in their abilities to express their own ideas with others, to work supportively, and to manage interpersonal conflicts. The instrument consists of 15 items assessing the level of confidence in different social situations (e.g., “Put yourself in a new and different social situation” and “Find someone to go to lunch with”). Responses receive a score from 1 (“no confidence at all”) to 5 (“complete confidence”). Scores of the instrument are calculated by adding up the scores of each item (Cronbach’s alpha value is 0.81).

#### The Hypercompetitive Attitude Scale

The Hypercompetitive Attitude Scale (HCA; Ryckman et al., 1997; Menesini et al., 2018) consists of 26 items assessing individual differences in hypercompetitive attitudes (e.g., “Winning in competition make me feel more powerful as a person” and “It’s a dog-eat-dog world. If you don’t get the better of others, they will surely get the better of you”). Responses receive a score from 1 “never true of me” to 5 “always true of me.” Higher scores refer to a stronger HCA. Cronbach’s alpha value is 0.78.

#### The Coping Strategy Indicator

The Coping Strategy Indicator (CSI; Nigro, 1996) is a self-report questionnaire on the degree to which the coping strategies of problem-solving (e.g., Have you tried to make a detailed plan of action rather than act on impulse? Cronbach’s alpha = 0.80), avoidance or avoiding events (e.g., Have you tried to distract yourself from the problem? Cronbach’s alpha = 0.79), and seeking social support (e.g., Did you accept help from a friend or relative? Cronbach’s alpha = 0.76) have been employed to cope with a specific stressor. It consists of 33 items measured on a 3-point scale with three subscales of 11 items each.

### Analysis Plan

First, we divided the participants into three groups based on the scores they reported on the SOGS (occasional gamblers: 0–2 points, problem gamblers: 3–4 points, pathological gamblers: from 5 points onward). Therefore, we conducted analyses of variance with *post hoc* tests. Subsequently, we conducted a linear regression in which we included hypercompetitiveness, self-efficacy, creativity (complexity + curiosity), and coping strategies as independent variables and the total mean scores that the participants reported on the SOGS as a dependent variable. In this way, we were able to test the risk and protective factors connected to gambling.

## Results

To evaluate any differences between groups of occasional gamblers, problem gamblers, and pathological gamblers, we conducted the ANOVA and *post hoc* comparisons (LSD) (Table 1). From the analyses, statistically significant differences emerged regarding coping avoidance strategies, *F*(2, 272) = 5.81, *p* < 0.01: the pathological gamblers scored higher [*M* = 22.65, *SD* = 3.5, MD (IJ) = 2.06, *p* < 0.05] than occasional gamblers [*M* = 20.59, *SD* = 4.4, MD (IJ) = −2.06, *p* < 0.05]; hypercompetitiveness, *F*(2, 272) = 11.21, *p* < 0.001: pathological gamblers reported significantly higher scores [*M* = 30.81, *SD* = 6.53, MD (IJ) = 4.15, *p* < 0.05] than both occasional gamblers (*M* = 26.66, *SD* = 5.9, MD (IJ) = −4.15, *p* < 0.05) and problem gamblers [*M* = 27.58, *SD* = 6.86, MD (IJ) = −3.23, *p* < 0.05]. Finally, statistically significant differences emerged in relation to the complexity factor of creativity, *F*(2, 271) = 3.03, *p* < 0.05, where problem gamblers showed higher scores [*M* = 26.54, *SD* = 3.12, MD (IJ) = 1.17, *p* < 0.05] than occasional gamblers [*M* = 25.37, *SD* = 3.2, MD (IJ) = −1.17, *p* < 0.05].

**TABLE 1:** Descriptive statistics on different groups of PGs.

Variables	Occasional gamblers	Problem gamblers	Pathological gamblers	*F*(2, 272)	*p*
	(*n* = 82)	(*n* = 80)	(*n* = 110)		
	*Mean* (*SD*)	*Mean* (*SD*)	*Mean* (*SD*)		
Seeking social support	22.85 (5.16)	22.74 (5.62)	22.64 (5.17)	0.04	0.96
Problem solving	24.40 (5.24)	24.48 (5.24)	24.92 (4.42)	0.32	0.72
Avoidance	20.59 (4.41)*	21.55 (4.70)	22.65 (3.52)*	**5.81**	0.003*
Social self-efficacy	50.04 (11.2)	51.09 (8.6)	48.11 (10.48)	2.10	0.15
Curiosity	26.95 (3.2)	27.79 (3.72)	27.25 (3.53)	1.10	0.31
Complexity	25.37 (3.2)*	26.54 (3.12)*	26.14 (2.98)	**3.03**	0.05*
Hyper-competitiveness	26.66 (5.9)*	27.58 (6.85)*	30.81 (6.53)*	**11.21**	0.00**

*p < 0.05; **p < 0.01. The bold values are statistically significant values.

To further examine our data set, we conducted a linear regression model analysis to verify which variables among coping strategies, perceived social self-efficacy, creativity, and hyper-competitiveness were predictors of gambling. The model is significant, *F*(2, 267) = 7.60, *p* < 0.001, *R*^2^ = 15) (Table 2). In particular, it suggested that the factors significantly connected to gambling were high levels of avoidance coping strategy (ß = 0.13, *SE* = 0.04, *p* < 0.05), high levels of the hypercompetitive attitude (ß = 0.24, *SE* = 0.023, *p* < 0.001), and low levels of perceived social self-efficacy (ß = −0.12, *SE* = 0.02, *p* < 0.05). Age was a control variable in this model without showing any significant effect.

**TABLE 2:** Summary model with PG predictors.

Variables	ß	SE	T	SIGN. (*P*)
Creativity (CU + CO)	0.03	0.021	0.51	0.60
Hyper-competitiveness	**0.24**	**0.023**	**4.17**	0.00***
Seeking social support	−0.09	0.03	−1.4	0.16
Problem-solving	0.04	0.03	0.55	0.58
Avoidance	**0.15**	**0.04**	**2.30**	0.02*
Self-efficacy	−**0.12**	**0.02**	−**2.15**	0.03*

*p < 0.05; **p < 0.01; ***p < 0.001. The bold values are statistically significant values.

## Discussion and Conclusion

The aim of this exploratory study was to find a framework of defending and risking factors connected to the genesis of gambling in a sample of teenagers as well as to highlight the peculiarities of regular gamblers. From the analyses conducted on male adolescents involved in the study, it emerged that pathological gamblers manifest higher levels of coping avoidance strategies, especially in comparison with occasional gamblers. It is likely that an increase in gambling frequency would produce gamblers’ troubles intensification, such as economic issues and relational and social problems, which cause the adolescent gambler’s need to avoid and disregard those complications. Therefore, the use of avoidant coping strategies in pathological gamblers may signify efforts to fight off stressful events through disavowal (Gupta et al., 2004; Bergevin et al., 2006; Shead et al., 2010). Similarly, as they are now excessively involved in or even addicted to the compulsive behavior of gambling itself and because addictive behaviors affect social spheres and interpersonal relationships, pathological gamblers become at risk of social isolation, not considering the outside world as a resource to solve complex and problematic situations.

Moreover, our findings underline differences concerning occasional gamblers and problem gamblers regarding the “complexity” factor of the creative personality (Pascual-Leone et al., 2011). Adolescent problem gamblers scored higher than occasional gamblers on this adaptive factor, probably because they are not yet addicted to gambling. This result may be explicated by the fact that adolescents who are more creative and who like to solve tasks with complex scenarios are also those who are more likely to gamble, but only up to a certain level of risk. Moreover, what this finding likely shows is that problem gamblers perceive themselves as being more complex thinkers than occasional gamblers, in line with the fact that, in much research, PG has been found to be related to several cognitive illusions and distortions (e.g., Johansson et al., 2009; Passanisi et al., 2017).

This study also underlines pathological gamblers reporting higher levels of hypercompetitiveness than the other considered groups (Burger et al., 2006). In other words, these players, being now victims of the compulsive, unconstrained cycle of addictive behaviors (Perales et al., 2020), have developed a greater tendency to be hypercompetitive because, even unknowingly, they always want to have more, reach the maximum by challenging the group, thinking they are the best. Furthermore, according to Chantal et al. (1994), highly competitive persons are more intrinsically interested and more prone to use a greater emotional involvement and quantity of time in gambling actions than persons who are extrinsically motivated by money to engage in gambling behaviors. Thus, this excessive level of involvement between highly competitive people may result in a greater level of problem gambling.

In line with the literature, our finding underlines the strength of the model of joint risk and protective factors with a lack of perceived social self-efficacy (Jeong and Kim, 2011) and high levels of both coping avoidance (Bergevin et al., 2006) and hypercompetitiveness connected to gambling (Burger et al., 2006). Hypercompetitiveness may be considered a risk factor as it can cause the male teenager to implement an addictive behavior only to show others and himself that he is the strongest of the group, that he can take the risk, he can make it and win. Even if satisfying for the player, this can lead to the cycle of gambling addiction because the adolescent can easily lose contact with the reality of his possibilities. Further, poor perceived social self-efficacy can contribute to increasing the risk of PG because an adolescent who does not feel capable in his social skills might find the answer to his discomfort in the game. In this context, gambling becomes a maladaptive response to an adaptive need that is lacking. Finally, the excessive employment of an avoidance coping strategy, in accordance with massive research (Bergevin et al., 2006; Casey et al., 2017), may lead to an attitude of closure toward the outside world and to a socio-affective immaturity, resulting in regular gambling activities. Avoidance, a distinctive characteristic of addicted individuals (Verner-Filion et al., 2014), may make adolescents incapable of managing emotions at a social-cognitive level, consequently finding an apparent containment in gambling activities.

Although the study extends the reference literature, it must be considered in light of its limitations: First, the use of self-report questionnaires could provide information that is not pure because answers might be affected by social desirability. Future studies could, for instance, make use of structured interviews and other clinical measures.

Second, the present study did not consider the relevance of findings regarding impulsivity (e.g., Grant et al., 2016; Passanisi and Pace, 2017; Pace and Passanisi, 2018) and cognitive biases, for instance, magical thinking (Johansson et al., 2009; Passanisi et al., 2017), that also play a role in PG and that the authors as well as other scholars better explored in past research with a main focus on young adults and adolescents where gambling is usually not yet a structured disorder.

A third constraint is that the study was conducted among a male adolescent regular gambler sample, so generalizability of results is limited. Future studies may be conducted with female participants to test the present model on gambling behaviors with regard to the other gender.

Another limitation is the cross-sectional nature of the current research. Future research, indeed, could verify this model in a multi-time perspective as well as including family and social variables that can be configured as risk and/or protective factors for the etiology of gambling difficulties. Finally, a longitudinal perspective would enable a better exploration, through adolescent development, of the individual differences in terms of both cognitive and personality variables, mainly in adolescents at risk of behavioral addictions, such as regular gamblers, to inform good practice and prevention programs.

## Data Availability Statement

Any information about the data can be requested directly from the corresponding author.

## Ethics Statement

The studies involving human participants were reviewed and approved by the Internal Review Board for Psychological Research of the UKE – Kore University of Enna. The patients/participants provided their written informed consent to participate in this study.

## Author Contributions

AP made contributions to conception and design, gathered and analyzed the data, was involved in drafting the manuscript, revised it and approved the final version to be published. GD’U made analysis and interpretation of data and drafted the manuscript. AS revised the manuscript, gave final approval of the version to be published, and agreed to be accountable for all aspects of the work in ensuring that questions related to the accuracy or integrity of any part of the work were appropriately investigated and resolved. SR revised the manuscript and agreed to be accountable for all aspects of the work. UP contributed to the first conception and design of the study, made analysis and interpretation of data, and drafted and revised the manuscript. All authors contributed to the article and approved the submitted version.

## Conflict of Interest

The authors declare that the research was conducted in the absence of any commercial or financial relationships that could be construed as a potential conflict of interest.
